# Supplementary material for: Long-term SARS-CoV-2 RNA shedding and its temporal association to IgG seropositivity
Source: Cell Death Discov. 2020 Dec 2;6:138. doi: 10.1038/s41420-020-00375-y (PMC7709096; doi:10.1038/s41420-020-00375-y)
Supplement: Supplementary file 1 — Supplementary figure legend [file 41420_2020_375_MOESM1_ESM.docx]

**Supplementary Figure Legends**

**Figure 4 – Supplementary Figure 1.** Extrapolating the distribution of percentage of positive culture values from PCR Ct values, for: **(a)** all tests from 208 COVID-19 patients, **(b)** tests conducted after 21 days of initial diagnosis, **(c)** tests conducted after 28 days of initial diagnosis.
