# Supplementary figures and images for: Long-term SARS-CoV-2 RNA shedding and its temporal association to IgG seropositivity
Source: Cell Death Discov. 2020 Dec 2;6:138. doi: 10.1038/s41420-020-00375-y (PMC7709096; doi:10.1038/s41420-020-00375-y)

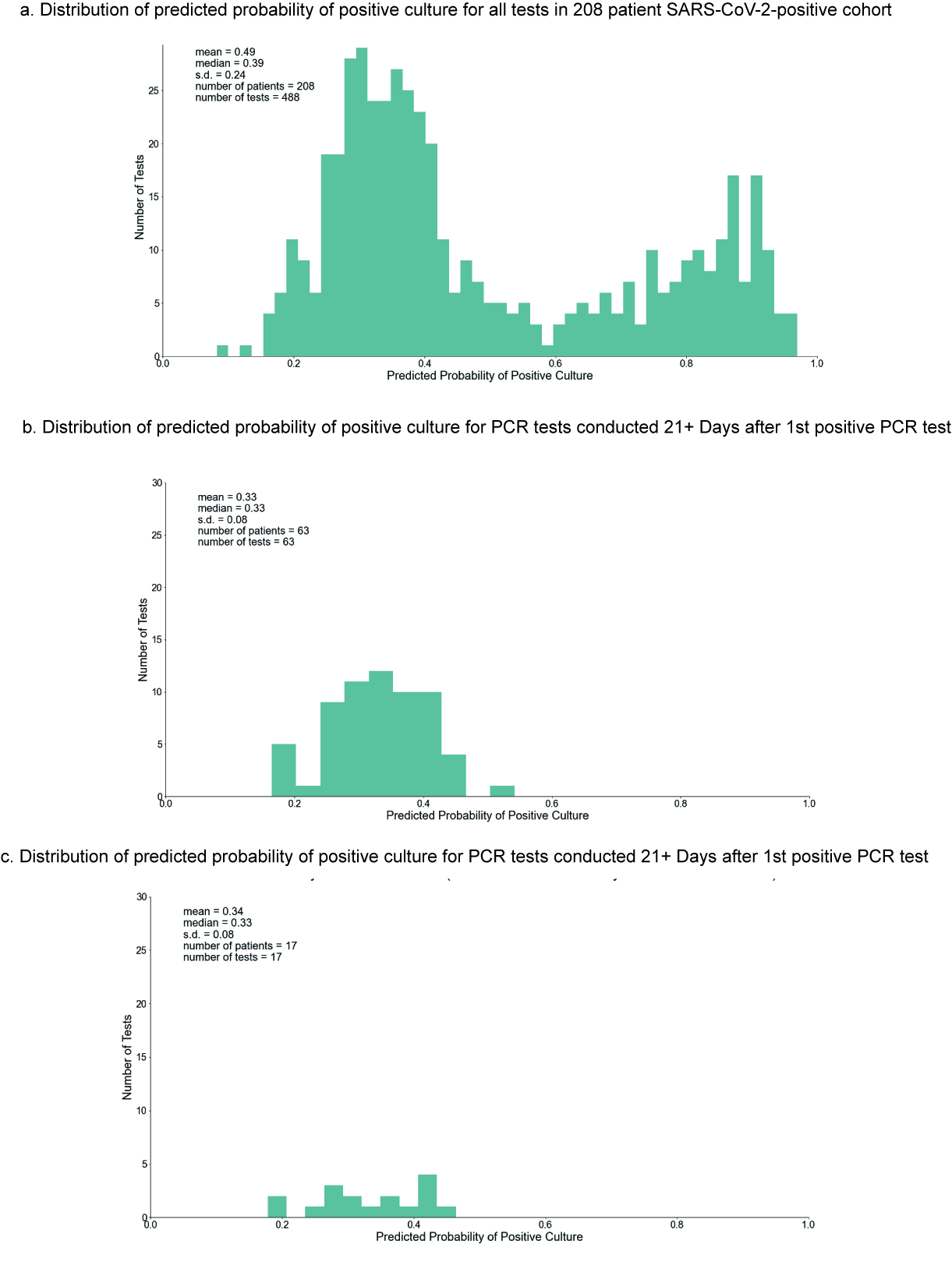

Supplement: Supplementary file 2 — Supplementary figure 4 - Part 1 [file 41420_2020_375_MOESM2_ESM.tif]
